# An efficient smoothing algorithm for range external guidance data based on dynamic threshold and adaptive interpolation

**DOI:** 10.1038/s41598-025-99382-1

**Published:** 2025-04-29

**Authors:** Shixue Zhang, Huihui Cai, Houfeng Wang, Shuai Liu, Qiang He

**Affiliations:** 1 Changchun Institute of Optics, Fine Mechanics and Physics, Chinese Academy of Sciences, Changchun, 130033 China; 2 University of Chinese Academy of Sciences, Beijing, 100039 China; 3 Changchun College of Electronic Technology, Changchun, 130022 China

**Keywords:** Photoelectric theodolite, Range measurement and control, External guidance data, Outlier detection, Dynamic threshold, Adaptive interpolation, Optics and photonics, Optical techniques

## Abstract

To achieve real-time smoothing of external guidance data for photoelectric theodolites and ensure stable image acquisition, this paper proposes a field processing method based on dynamic thresholding and adaptive interpolation. First, to address outliers in the external guidance data, a method for dynamically calculating sample variance using an influence function is introduced. This approach establishes an adaptive threshold for real-time outlier detection in real time. Second, for the interpolation of external guidance data, the coherence of the interpolation calculations is evaluated, and the “stuck” external guidance data is categorized. Different strategies are applied based on the severity of the stuck condition, enabling real-time smooth interpolation. Experimental results demonstrate that the proposed dynamic threshold-based five-point extrapolation outlier elimination method achieves an average outlier detection rate of over 80% with a very low false alarm rate. The interpolation algorithm effectively handles both stuck and non-stuck external guidance data, producing smooth and coherent processing results. This method has demonstrated significant results in practical application, meeting the requirements of the trajectory measurement system.

## Introduction

The photoelectric theodolite offers several advantages, including stable performance, high precision, and strong anti-interference capabilities^1,2^. In range experiment tasks, multiple photoelectric theodolites are often employed to perform intersection measurements of the same aircraft target. The primary process involves target acquisition, encoder data generation, data transmission, data reception, and intersection calculation^3^. This process ultimately yields accurate ballistic parameters, such as trajectory, which necessitates that each photoelectric theodolite in the measurement and control system provides timely and precise measurement data. As an angle-measuring instrument, the photoelectric theodolite typically has a narrow field of view in its telephoto lens, making it challenging to capture targets independently through manual control alone^4,5^. In the external guidance tracking mode, the central station transmits real-time target position information to the photoelectric theodolite, enabling target acquisition within the field of view. Moreover, when the target cannot be obtained in the image closed-loop tracking mode^6^, the external guidance mode ensures the continuity of the tracking process. Once image interpretation and target extraction stabilize, the system can switch back to the image closed-loop tracking mode. Consequently, the external guidance mode plays a critical role in the operational workflow of the theodolite^7^.

The real-time measurement of the target’s azimuth and elevation angles is fundamental to the theodolite post-processing system and the overall experimental task. In the external guidance mode, inaccurate or unsmoothed guidance information, as well as unstable image acquisition, can compromise the accuracy of trajectory calculations for space targets^6,8^. Therefore, it is essential to develop a real-time interpolation algorithm for external guidance data to ensure its accuracy and smoothness. External guidance data can be acquired from various measurement systems, such as telemetry and radar^9^. However, due to environmental interference, equipment accuracy limitations, and other factors, the external guidance data often contains outliers^10^. Additionally, the external data frequency is typically lower than the self-guidance frequency of the photoelectric theodolite. Consequently, the smoothing algorithm must detect and eliminate outliers^11,12^ while generating interpolated data at an appropriate frame rate to serve as reliable guidance data for the photoelectric theodolite.

For other fields such as UAV control, considering the impact of sensor uncertainty on flight control, the researchers developed an algorithm to adjust the control strategy online to cope with sensor noise and uncertainty, thus significantly improving the autonomous flight ability and trajectory tracking accuracy of the UAV. Although these methods have achieved remarkable results in their respective fields, it is often difficult for the fixed adjustment strategy of parameters to achieve the ideal smoothing effect in the external guided data processing of photoelectric theodolite.

In the field of outlier elimination, numerous scholars have conducted extensive research. Outliers can be categorized into two types based on their occurrence patterns: isolated outliers and continuous outliers^13^. Reference^14^ provides an experimental analysis of several common outlier processing methods currently used in engineering applications. Among these, the polynomial extrapolation fitting method is characterized by its simplicity and effectiveness in eliminating isolated outliers. However, its performance in handling continuous outliers is less satisfactory, and determining an appropriate discrimination threshold remains challenging. Reference^15^ proposed a wavelet denoising algorithm for outlier elimination, which demonstrates good performance but is unsuitable for real-time processing scenarios. Reference^16^ introduced a grey prediction-based method for adaptive outlier detection, which also struggles to achieve satisfactory results for continuous outliers.

In terms of interpolation processing, the most commonly used algorithms in range measurement are linear least squares interpolation and Newton interpolation. However, during actual tasks, we observed that external guidance data often experiences a “stuck” situation, where consecutive frames of external guidance data retain the same value. In such cases, conventional interpolation algorithms fail to ensure smoothness, which can lead to abrupt movements and frame jitter during the photoelectric theodolite’s tracking process. This issue may severely damage the servo system. Currently, linear interpolation is predominantly used in range measurement and control^17,18^. However, there is limited research on smoothing methods specifically designed to address “stuck” data in external guidance.

To address the aforementioned issues, this paper proposes an external guidance smoothing method, which consists of a dynamic threshold-based outlier processing algorithm and an adaptive interpolation algorithm.

1. *Outlier processing algorithm*: (1) The unified form of sample variance calculation is obtained by using the influence function. (2) It is proposed to solve the unknown parameters by using the truncated normal distribution, thereby constructing the dynamic threshold. This algorithm addresses the problem of inaccurate sample variance estimation when sample points contain outliers and achieves superior detection rates and lower false alarm rates compared to fixed-threshold methods;
2. *Interpolation algorithm*: (1) By introducing two coherence judgments, the coherence relationship between the external guide received value, the interpolation data of the previous time and the linear least square method is obtained. (2) Varying interpolation strategies are employed based on the classification of the received external guidance value. This algorithm effectively handles discontinuities caused by “stuck” external guidance data. It adaptively calculates interpolation points based on the characteristics of the external guidance data, ensuring smooth and coherent results.

The proposed smoothing algorithm has been successfully implemented in a naval measurement system, delivering satisfactory performance.

## Outlier processing based on dynamic threshold

In exterior ballistic measurement, outlier processing methods can be broadly categorized into three types. The first type relies on analyzing the statistical characteristics of the data. Outliers are detected and eliminated by calculating the standard deviation and setting a reasonable threshold, as exemplified by the Wright criterion and Romanovsky criterion^17^. The second type involves distinguishing and eliminating outliers by extracting and analyzing data features, such as through wavelet transform^14–18^. The third type focuses on obtaining and analyzing the statistical characteristics of the difference between the filtered estimated values and the original data values for outlier discrimination^19–21^.

In engineering applications, a commonly used real-time outlier processing algorithm involves utilizing data points from previous time steps to calculate an extrapolated value and construct a threshold. If the difference between the extrapolated value and the currently received value exceeds this threshold, the received value is identified as an outlier and replaced with the extrapolated value. This method has low complexity and is widely used in engineering. However, the data used to calculate sample variance may contain wild values, resulting in inaccurate variance estimation. Consequently, data extrapolation and threshold construction are critical components of outlier processing. To address the challenges of determining an accurate and reliable outlier discrimination threshold, we propose a method that calculates sample variance using an influence function and constructs a dynamic threshold.

### Common outlier processing algorithms

When selecting the five data points preceding the current time, the five-point extrapolation formula is expressed as Formula (1). The current time is denoted as $y_k$, and the five preceding time points are $y_{k-5}$, $y_{k-4}$,…, $y_{k-1},$ respectively.

1 $$\begin{aligned} \hat{y}_k = -\frac{2}{5}y_{k-5} - \frac{1}{10}y_{k-4} + \frac{1}{5}y_{k-3} + \frac{1}{2}y_{k-2} + \frac{4}{5}y_{k-1} \end{aligned}$$

According to Wright’s criterion, when data follows a normal distribution, the residuals between the data points and the sample mean predominantly lie within three times the standard deviation, with the probability of exceeding this range being no more than 0.3%^22^. Assuming that the residuals $\Delta y_k$ between the external guidance data $y_k$ transmitted at time *k* and the five-point extrapolation data follow a normal distribution with a mean of 0^23^, the sample standard deviation of $\Delta y_k$ is denoted as $\hat{\sigma }$.

The criteria for handling outliers are as follows:

- When $|\Delta y_k < 3\hat{\sigma }|$, $y_k$ is a normal value and will not be processed;
- When $|\Delta y_k \ge 3\hat{\sigma }|$, $y_k$ is an outlier value, $y_k$ is replaced with $\hat{y}_k$ .

At each time step, we calculate the five-point extrapolation value $\hat{y}_i$ and the residual value $\Delta y_i$. Based on the current time, a sliding window of length *n* is established. The measurement data sequence within the window is denoted as $\{y_i\}$, and the extrapolation data sequence as $\{\hat{y}_i\}$. Consequently, the residual sequence is $\{\Delta y_i\}$, where $\Delta y_i = y_i - \hat{y}_i$. Using Formula (2), we can compute three times the sample standard deviation.

2 $$\begin{aligned} \hat{\sigma }^2 = \frac{1}{n-1}\sum _{i=k-n}^{k-1}(\Delta y_i)^2 \end{aligned}$$

### Outlier processing algorithm based on dynamic threshold

During the process of tracking and measurement, the data points within the sliding window may exhibit the presence of outliers, especially in the form of continuous outliers. In these instances, the consecutive sampling points preceding and succeeding the outliers may also be influenced. Consequently, the sample variance computed using Formula (2) may not accurately represent the variability of the data within the sliding window. This discrepancy can result in an unreliable estimation of the sample variance, thereby diminishing the precision of the discrimination threshold. Therefore, it is imperative to enhance the methodology employed for calculating the sample variance.

Assuming that there is a prior standard deviation $\sigma$ of the known data, we also specify the threshold value $C_H$, and the rules for distinguishing the data in the sliding window from normal or outlier values are as follows:

Assuming a prior standard deviation $\sigma$ of the known data, we also define the threshold value $C_H$. The criteria for differentiating between normal and outlier values within the sliding window are established as follows:

- if $|\Delta y_i/\sigma |<C_H$, $y_i$ is normal;
- if $|\Delta y_i/\sigma |\ge C_H$, $y_i$ is an outlier.

It is essential to emphasize that the prior standard deviation is utilized exclusively to assess the normality or abnormality of the data within the sliding window, rather than being directly applied as the sample standard deviation for the purpose of identifying outliers.

A novel formula for the computation of sample variance has been developed utilizing the influence function, as demonstrated in Formula (3).

3 $$\begin{aligned} \sum _{i=k-n}^{k-1}\psi ^2(\Delta y_i/\hat{\sigma }) = (n-1)\beta \end{aligned}$$

Here, $\psi (x)$ represents the influence function, $\hat{\sigma }$ is the sample standard deviation to be determined, and $\beta$ is an undetermined parameter. The influence function is used to differentiate between normal and abnormal data points within the sliding window, enabling the calculation of a more accurate sample variance based on the normal data points.

We have chosen Huber’s function, represented as $\psi _H(x)$, to serve as the influence function^24^. This function is characterized by its piecewise definition.

4 $$\begin{aligned} \psi (x) = {\left\{ \begin{array}{ll} x & \text { if } |x| < C_H \\ C_HSign(x) & \text { if } |x| \ge C_H \end{array}\right. } \end{aligned}$$

In Formula (3), the parameter $\hat{\sigma }$ is not known, and thus the function $\psi (\Delta y_i/\hat{\sigma })$ cannot be computed directly during the analysis. Only the normal point within the sliding window can reliably represent the sample variance. Therefore, the processing methodology, which differentiates between normal and abnormal values, is outlined in Formula (5).

5 $$\begin{aligned} \psi ^2(\Delta y_i/\hat{\sigma }) = {\left\{ \begin{array}{ll} (\Delta y_i)^2/\sigma ^2 & \text { if } |\Delta y_i/\sigma | < C_H \\ C^2_H & \text { if } |\Delta y_i/\sigma | \ge C_H \end{array}\right. } \end{aligned}$$

We define $M_n$ as the collection of normal value points within the sliding window, and $N_H$ as the number of abnormal value points within the same window. By replacing Eq. (5) with Eq. (3), the adaptive sample variance is then expressed in Eq. (6).

6 $$\begin{aligned} \begin{aligned} \hat{\sigma }^2 = \textstyle \sum _{M_n}(\Delta y_i)^2 / d\\ d = (n-1)\beta - N_HC^2_H \end{aligned} \end{aligned}$$

The prior threshold $C_H$ and the prior standard deviation $\sigma$ are predetermined values; consequently, the key focus of variance estimation is the calculation of the parameters $\beta$, which can be articulated as demonstrated in Formula (7).

7 $$\begin{aligned} \beta = \frac{1}{n-1} \left[ \textstyle \sum _{M_n}(\Delta y_i/\sigma _{cut})^2(\sigma _{cut}/\sigma )^2 + N_HC^2_H \right] \end{aligned}$$

Generally, outliers are not expected to significantly alter the distribution characteristics of $\Delta y_i$ within the sliding window. It is assumed that the residuals adhere to a normal distribution with a mean of 0. The standard deviation of this normal distribution is designated as a predetermined standard deviation, such that $\Delta y_i \sim \mathcal {N}(0, \sigma ^2)$, and consequently, $\Delta y_i/\sigma \sim \mathcal {N}(0, 1)$.

It is established that the normal value points are defined as those that satisfy the condition $|\Delta y_i/\sigma | < C_H$ within a sliding window. The corresponding values of $\Delta y_i/\sigma$ adhere to a standard normal distribution, indicating that the normal value points follow a truncated normal distribution^25^. Specifically, this can be expressed as $\Delta y_i/\sigma \sim \mathcal {N}(0, 1; -C_H, C_H)$. The expected value and variance of this truncated normal distribution are denoted as $\mu _{cut}$ and $\sigma ^2_{cut}$, respectively, while the number of data points in the set $M_n$ is represented as $N_n$. In Eq. (2), the sample variance may also be interpreted as a substitution for the variance. In conclusion, the normal value points conform to the relationship articulated in Eq. (8).

8 $$\begin{aligned} \textstyle \sum _{M_n}(\Delta y_i/\sigma _{cut})^2 = N_n - 1 \end{aligned}$$

Substituting Formula (8) into Formula (7) can be written as shown in Formula (9).

9 $$\begin{aligned} \beta = \frac{N_n-1}{n-1}(\sigma _{cut}/\sigma )^2 + \frac{N_H}{n-1}C^2_H \end{aligned}$$

Both normal and abnormal value points conform to the relationship $\Delta y_i/\sigma \sim \mathcal {N}(0, 1)$, suggesting that the proportion of data points can be approximated by the corresponding distribution probabilities. Let $P_1$ denote the probability that the random variable $\Delta y_i/\sigma$ falls within the interval $[-C_H, C_H]$, while $P_2$ represents the probability of the distribution within the intervals $[- \infty , -C_H]$ and $[-C_H, +\infty ]$. Consequently, the expressions $(N_n-1)/(n-1)$ and $N_H/(n-1)$ can be respectively represented by $P_1$ and $P_2$. The probability distribution function of the standard normal distribution is denoted as $\Phi (x)$, with the calculation method detailed in Formula (10).

10 $$\begin{aligned} \frac{N_n-1}{n-1}=\Phi {C_H}-\Phi (-C_H)=P_1=1-P_2 \end{aligned}$$

According Eqs. (9) and (10), $\beta$ are calculated with the different corresponding $C_H$ values as shown in Table 1.

**Table 1:** $C_H$ and its corresponding $\beta$.

$C_H$	1.2	1.3	1.4	1.5	1.6	1.7
$\beta$	0.5149	0.5677	0.6179	0.6650	0.7087	0.7489

Specifically, as $C_H$ approaches infinity, $P_1$ converges to 1, and $\beta$ approaches 1, these conditions will affect the function $\psi (x) = x$. Consequently, Eq. (6) will reduce to Eq. (2). It can be posited that Eq. (6) serves as a generalized representation of sample variance, while Eq. (2) is regarded as a particular instance of Eq. (6).

The process of outlier algorithm is as follows:

1. Receive the external guidance data $y_k$ at the current time and calculate the five points extrapolation value $\hat{y}_k$ and residual error $\Delta y_k$;
2. Based on the residual sequence $\{\Delta y_i\}$ in the sliding window of above n points in the current time, count the number of outliers $N_H$;
3. Calculate the sample standard deviation $\hat{\sigma }$ according Eq. (6), and take $3\hat{\sigma }$ as the dynamic threshold;
4. According to the criterion of outliers, compare $|\Delta y_k|$ and $3\hat{\sigma }$ to complete the next process.

In the context of the experimental task, outliers are not identified when the absolute value of the residual is minimal. When a continuous set of external guidance data, such as 40 data points, is classified as outliers due to identical values, it may be interpreted as fixed-point guidance data from the theodolite. At this juncture, we reset the data variable and initiate a new round of detection and classification.

## Adaptive interpolation algorithm

The communication frequency of a specific range photoelectric theodolite is established at 100 Hz, whereas the external guidance data from the central station is limited to 20 Hz. Each time the interpolation algorithm receives a data point from the external guidance, it is required to generate five interpolated data points. Given that this interpolated external guidance data is directly utilized by the Theodolite Servo System, it is imperative that the data be smooth and continuous. When the theodolite rotates in a particular direction, any increase or decrease in the interpolated data must occur gradually; abrupt changes could significantly compromise the integrity of the Theodolite Servo System. To address these challenges, we propose the implementation of an adaptive interpolation algorithm.

### Common interpolation algorithms

#### Linear least square method

Let us denote the current time as *k*, at which we receive external guidance data at a frequency of 20 Hz, represented as $y_k$. We will consider the external guidance data from both the current and preceding time points, specifically selecting ten data points: $y_{k-9}, y_{k-8}, \ldots , y_k$. Utilizing these data points, we will formulate a linear equation based on the least squares method. This approach will enable us to derive five-point interpolation data at the time intervals $k-0.8, k-0.6, \ldots , k$, which will serve as the actual guidance data at a frequency of 100 Hz.

#### Newton interpolation

When the external guidance consists of equidistant nodes derived from fixed frequency data, the Newton interpolation method can be further streamlined. In this context, the forward interpolation formula^26^ serves as a pertinent example.

11 $$\begin{aligned} N_n(x_0+th)=f_0+t\Delta f_0+\frac{t(t-1)}{2!}\Delta ^2f_0+\cdots +\frac{t(t-1)\cdots (t-n+1)}{n!}\Delta ^nf_0 \end{aligned}$$

Where $$\Delta f_i = f_{i+1}-f_i$$, $$\Delta ^kf_i = \Delta ^{k-1}f_{i+1} - \Delta ^{k-1}f_i$$.

In the context of external guidance data for the range, a quadratic curve is employed to represent the shape of the curve, with *n* set to 2, $x_0$ designated as the current $y_k$, and *h* assigned a value of -1. The values $t = 0.8, 0.6, \ldots , 0$ will be substituted into Eq. (11) to derive the interpolation data corresponding to the time points $k-0.8, k-0.6, \ldots , k$.

### Stuck situation in external guide

**Fig. 1:**
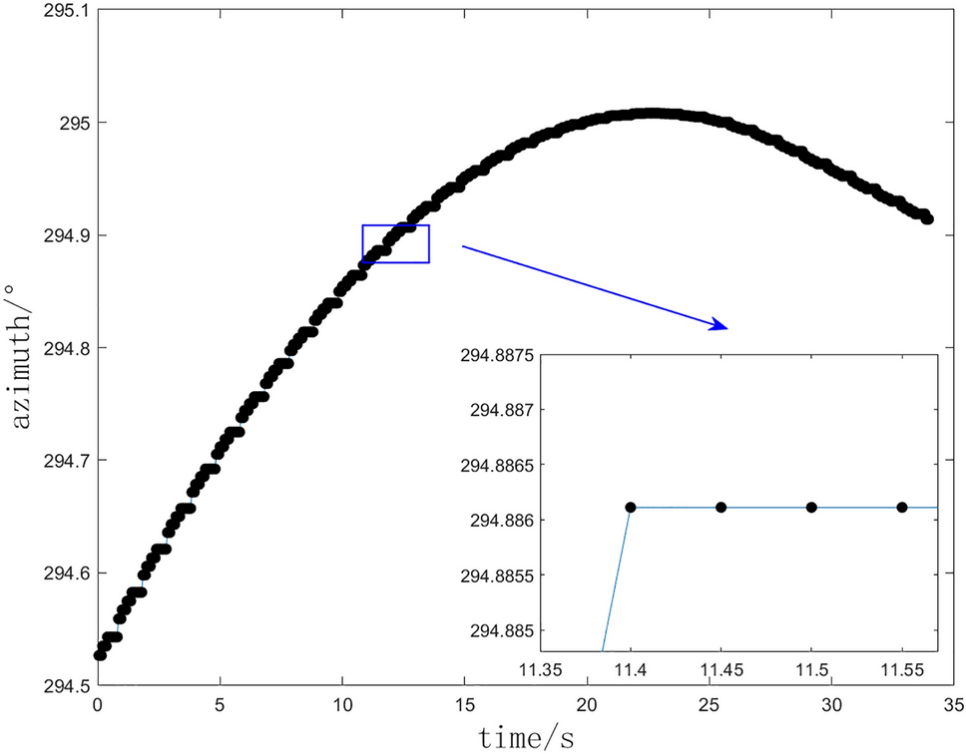
Stuck point and interpolation data.

During the outfield experiment, the equipment may encounter a phenomenon referred to as “stuck,” wherein it receives identical external guidance data for consecutive frames transmitted by the central station. This is illustrated in Fig. 1, which depicts the azimuth external guidance data received by the theodolite. The horizontal coordinate in Fig. 1 is the time point of the current sub-task data, and vertical ordinate is the azimuth guide angle value. The occurrence of this phenomenon is frequently attributed to a low and unstable sampling rate of the measurement source. Additionally, it may arise from the central station’s transition between data sources^27^, resulting in the external guidance data being unable to update promptly and remaining constant at the value from the previous transmission.

**Fig. 2:**
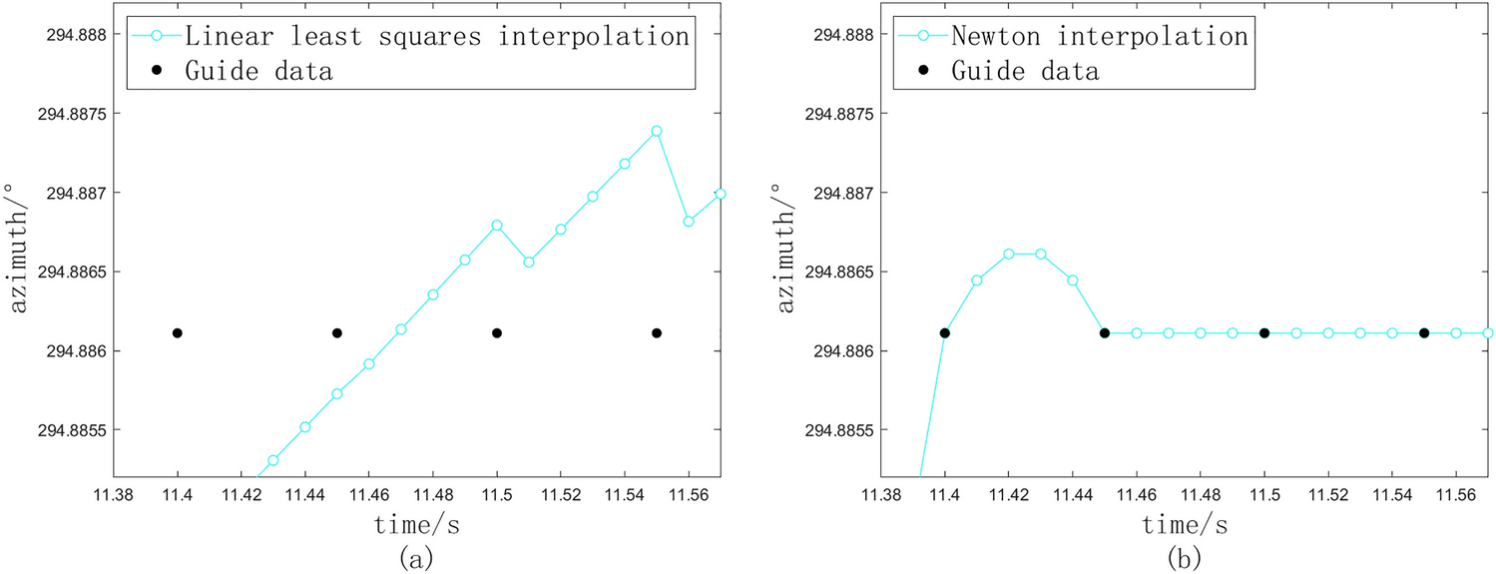
Stuck point and interpolation data. (**a**) Stuck situation. (**b**) Linear least squares interpolation. (**c**) Newton interpolation.

In the scenario of a stuck mechanism, the interpolation data derived from the linear least squares method and the Newton interpolation method are illustrated in Fig. 2a and b, respectively. The current azimuth angle of the theodolite, as depicted in the figures, exhibits an increasing trend, which the interpolation data should ideally reflect. However, the interpolation results produced by both methods demonstrate significant inconsistencies and pronounced fluctuations. Notably, the interpolation data points presented in the figures are occasionally lower than preceding points, which contradicts the established increasing trend. If these data points are utilized directly as control signals for the servo system, the theodolite is likely to experience rapid oscillations, resulting in frequent and abrupt reversals during the tracking process. Empirical observations indicate that such behavior can lead to jitter in the theodolite and may even compromise the integrity of the servo system.

The linear least squares method, while effective in capturing the overall trend of sample points during interpolation, exhibits inconsistencies when encountering stuck points, as evidenced by the five data points collected at various interpolation intervals. Conversely, the Newton interpolation method, although capable of constructing quadratic curves, relies on each newly acquired external guide point to formulate the difference quotient. This approach ensures that the interpolation polynomial intersects the interpolation nodes, resulting in arc segments with varying concavity along the curve. However, both linear least square method and Newton interpolation method are calculated using sample points in a fixed window, which leads to the inconsistency of interpolation points. Especially for Newton interpolation method, even if the quadratic equation can meet the trend of fitting curve, it cannot control its increase or decrease. Such fluctuations in increasing and decreasing trends can lead to significant instability in the theodolite readings. In light of the limitations inherent in both methods, this paper introduces a novel processing algorithm grounded in adaptive interpolation techniques.

### Adaptive interpolation algorithm

The objective of this study is to generate interpolated data at a frequency of 100Hz based on input data sampled at 20Hz in real-time. We establish the following definitions: the guidance data received at the current time is denoted as $y_k$, while the most recent ten data points are represented as $y_{k-9}, y_{k-8}, \ldots , y_k$. The five-point interpolation computed at the previous time is denoted as $z_{k-1.8}, z_{k-1.6}, \ldots , z_{k-1}$, and the current interpolation data to be calculated is represented as $z_{k-0.8}, z_{k-0.6}, \ldots , z_k$. A valid point is defined as a guidance data point that differs from the value recorded at the previous time, whereas a stuck point is one that remains unchanged from the previous guidance value. Stuck points are categorized into two types based on the number of consecutive stuck points, denoted as *L*: slight stuck points, where the number of current stuck points is less than or equal to *L*, and serious stuck points, where the number exceeds *L*. The value of *L* is determined empirically. In the case of a valid point, we update the variable *flag* to indicate the trend of the azimuth angle at that moment. If the angle value of the valid point exceeds the previous external guidance value, indicating an increase in the guidance angle, *flag* is assigned a value of 1; conversely, if the angle value is less than the previous guidance value, *flag* is assigned a value of -1. At a stuck point, the value of *flag* remains unchanged from its previous state.

The adaptive interpolation is derived through the application of the linear least squares method, utilizing a set of ten guiding data points to obtain the values *LS*[0], *LS*[1], *LS*[2], *LS*[3], and *LS*[4]. Furthermore, two consistency assessments are incorporated based on this foundation.

The initial step involves assessing the consistency of the current five-point interpolation data, derived from the linear least squares method, with the trend observed in the previous five-point interpolation data. For instance, when the variable $flag > 1$, the current external guidance indicates an upward trend, suggesting that the interpolation data obtained in the previous iteration should not exceed the current interpolation results. Specifically, this assessment entails evaluating the sign of the product $flag \times (LS[0] - z_{k-1})$. A negative outcome signifies that *LS*(0) is relatively inconsistent with $z_{k-1}$, thereby indicating that the values $LS[0], \ldots , LS[4]$ cannot be utilized directly as the final interpolation data. It is imperative to ensure that the interpolation data reflect the observed increasing trend.

The second criterion involves assessing the consistency of the currently received external guidance data, denoted as $y_k$, with the previously interpolated five-point data. For instance, when $flag > 1$, the interpolated data from the previous iteration should not exceed the current external guidance data. Specifically, this assessment entails evaluating the sign of the product $flag \times (y_k - z_{k-1})$. A negative result indicates that the prior interpolated data surpasses the current valid data point, suggesting that $y_k$ is inconsistent with $z_{k-1}$. Furthermore, to prevent the theodolite from rotating in the opposite direction, any adjustments made to the five points of the current interpolation must be limited to a small increment, which is governed by the parameter *increment*.

The adaptive interpolation algorithm under consideration utilizes the results obtained from the linear least squares method as an intermediary to assess consistency. The algorithm’s procedure is delineated based on the categorization of external guidance data points.

If $y_k$ is a valid point, the algorithm is as follows:

1. Update *flag*;
2. According to the linear least square method, *LS*[0],…, *LS*[4] is calculated by $y_{k-9}$, $y_{k-8},\ldots,$ $y_k$;
3. Judge the condition $flag\times (LS[0]-z_{k-1})<0$. If true, continue with step 4; Otherwise, *LS*[0],…, *LS*[4] can be set to $z_{k-0.8}$, $z_{k-0.6},\ldots$, $z_k$, and end the process;
4. Judge the condition $flag\times (y_k-z_{k-1})<0$. If true, $z_{k-i}=z_{k-1}+flag\times increment \times (1-i);$ otherwise, we select the interpolation data according to the distance between $y_k$ and *LS*[0], that is $z_{k-i}=z_{k-1}+flag \times |LS[0]-y_k|\times (1-i)$, where $i=$ 0.8, 0.6,…, 0.

In instances of minor stuck points, the initial assessment is employed to maintain the consistency of interpolation data across varying reception times. Upon receiving the stuck point, denoted as $z_{k-1}$, it is anticipated that this value will gradually surpass $y_k$. Consequently, a second assessment will not be conducted; however, it is crucial that the interpolation data corresponding to the stuck point does not significantly diverge from $y_k$ and ideally remains below the predetermined threshold. Failure to adhere to this threshold may result in the theodolite’s inability to accurately detect the intended target.

If $y_k$ is a slight stuck point, the algorithm flow is as follows:

1. According to the linear least square method, *LS*[0],…, *LS*[4] is calculated by $y_{k-9}$, $y_{k-8}$,…, $y_k$;
2. Judge the condition $flag\times (LS[0]-z_{k-1})<0$. If yes, continue to step 3; Otherwise, *LS*[0],…, *LS*[4] can be set to $z_{k-0.8}$, $z_{k-0.6}$,…, $z_k$, and end the process;
3. Judge the condition $|y_k-z_{k-1}|>threshold$. if true, $z_{k-i}=z_{k-1}+flag\times |LS[0]-y_k|\times (1-i)$; Otherwise, $z_{k-i}=z_{k-1}+flag \times increment \times (1-i)$, where $i=$ 0.8, 0.6,…, 0.

In instances of significant stuck points, the valid data points can effectively represent the fundamental trends in azimuth and elevation. Consequently, three nearest valid points are selected as feature points for the external guidance data, in addition to three stuck points. Collectively, these six points are utilized to construct a linear least squares five-point interpolation dataset, which captures the underlying curve trend. It is imperative that the interpolation point, in cases of severe stasis, does not deviate excessively from the received external guidance (which remains the stuck point at this juncture). Therefore, an assessment is made to determine whether the value exceeds a specified threshold. The subsequent steps follow the previously outlined procedures and will not be elaborated upon further.

If $y_k$ is a serious stuck point, the algorithm flow is as follows:

1. Select the three valid points closest to the current receiving external guidance point, plus the three nearest stuck points $y_{k-2}$, $y_{k-1}$, $y_k$ to construct the linear least squares five points interpolation data, and the calculation result is still recorded as *LS*[0],…, *LS*[4].
2. Calculate the slope of the line on *LS*[0],…, *LS*[4], and record as *slope*.
3. Judge the condition $flag \times (LS[0]-z_{k-1})<0$. If yes, continue with step 4; Otherwise, *LS*[0],…, *LS*[4] can be set to $z_{k-0.8}$, $z_{k-0.6}$,…, $z_k$, and end the process.
4. Judge the condition $|y_k-z_{k-1}|>threshold$. If yes, we select interpolation data along the line with a slope of value , that is $z_{k-i}=z_{k-1} + slope \times (1-i)$; Otherwise $z_{k-i}=z_{k-1} + flag \times increment \times (1-i),$ where $i=$ 0.8, 0.6,…, 0.

The underlying mathematical framework of the adaptive interpolation algorithm parallels that of the linear least squares method; however, the latter fails to account for the correlation between successive interpolations as well as the relationship between the interpolated values and the observed data. In contrast, the adaptive interpolation algorithm ensures the coherence of two consecutive interpolation values, that is, the consecutive interpolation points keep rotating towards the same method, so that it is aligned with the overall trend of the guidance data.

The algorithm pseudo-code is as follows:

**Initialize parameters:**

01. flag $\leftarrow$ 0 // Trend indicator (1: increasing, -1: decreasing)

02. L $\leftarrow$ 4 // Threshold for slight stuck points

03. threshold $\leftarrow$ 0.2 // Data consistency threshold

04. increment $\leftarrow$ 0.0005 // Adjustment step size

05. stuck_count $\leftarrow$ 0 // Counter for consecutive stuck points

06.**Function adaptive_interpolation(y_k, history_data):**

07. ***Input:***

08.      **y_k**: Current external guidance data

09.      **history_data**: Historical data sequence (latest data points)

10. ***Output:***

11.      **z_new**: Generated interpolation data (5 points)

12. // Determine data point type

13. If $y\_k \ne history\_data[-1]$ (latest historical data):

14.      // Process valid point

15.      stuck_count $\leftarrow$ 0 // Reset stuck counter

16.      flag $\leftarrow$ 1 if y_k > history_data[-1], else -1 // Update trend indicator

17.      LS $\leftarrow$ Perform linear least squares interpolation using the latest 10 data points from history_data

18.      // Consistency check 1: Interpolation trend vs. historical trend

19.      if flag $\times$ (LS[first interpolated point] - previous interpolation end point) < 0:

20.           // Adjust interpolation based on conditions

21.           if flag $\times$ (y_k - previous interpolation end point) < 0:

22.                // Generate interpolation using fixed step size

23.                For each interpolation position $i \in {0.8, 0.6, 0.4, 0.2, 0}$:

24.                     z_new[i] $\leftarrow$ previous interpolation end point + flag $\times$ increment $\times$ (1 - i)

25.           else:

26.                // Generate interpolation using delta between LS and current value

27.                delta $\leftarrow$ |LS[first interpolated point] - y_k|

28.                For each interpolation position $i \in {0.8, 0.6, 0.4, 0.2, 0}$:

29.                     z_new[i] $\leftarrow$ previous interpolation end point + flag $\times$ delta $\times$ (1 - i)

30.      else:

31.           z_new $\leftarrow$ LS // Use LS results directly

32. else:

33.      // Process stuck point

34.      stuck_count $$\leftarrow$$ stuck_count + 1

35.      if $$stuck\_count \le L$$:

36.           // Slight stuck point

37.           LS $\leftarrow$ Perform linear least squares interpolation using latest 10 data points

38.           if flag $\times$ (LS[first interpolated point] - previous interpolation end point) < 0:

39.                if |y_k - previous interpolation end point| > threshold:

40.                     delta $\leftarrow$ |LS[first interpolated point] - y_k|

41.                     For each interpolation position $i \in {0.8, 0.6, 0.4, 0.2, 0}$:

42.                          z_new[i] $\leftarrow$ previous interpolation end point + flag $\times$ delta $\times$ (1 - i)

43.                else:

44.                     For each interpolation position $i \in {0.8, 0.6, 0.4, 0.2, 0}$:

45.                          z_new[i] $\leftarrow$ previous interpolation end point + flag $\times$ increment $\times$ (1 - i)

46.           else:

47.                z_new $$\leftarrow$$ LS

48.      else:

49.           // Severe stuck point

50.           valid_points $\leftarrow$ Select last 3 valid points from history_data

51.           stuck_points $\leftarrow$ Select last 3 stuck points (including current point)

52.           LS $\leftarrow$ Perform linear least squares interpolation using valid_points + stuck_points

53.           slope $\leftarrow$ Calculate slope of LS

54.           if flag $\times$ (LS[first interpolated point] - previous interpolation end point) < 0:

55.                if |y_k - previous interpolation end point| > threshold:

56.                     For each interpolation position $i \in {0.8, 0.6, 0.4, 0.2, 0}$:

57.                          z_new[i] $\leftarrow$ previous interpolation end point + slope $\times$ (1 - i)

58.                else:

59.                     For each interpolation position $i \in {0.8, 0.6, 0.4, 0.2, 0}$:

60.                          z_new[i] $\leftarrow$ previous interpolation end point + flag $\times$ increment $\times$ (1 - i)

61.           else:

62.                z_new $$\leftarrow$$ LS

63.      Return z_new

## Experimental results and analysis

### Outlier processing algorithm

#### Experimental setup

The investigation into the outlier processing algorithm encompasses both simulation experiments and real-world field experiments, with the guiding data consisting of the angle values obtained following coordinate transformation.

In the conducted simulation experiment, we selected a segment of genuine outfield test data that was devoid of outliers. Subsequently, we introduced several outliers and employed three distinct outlier processing methodologies: dynamic threshold, fixed threshold, and non-impact function. The fixed threshold approach calculates the adaptive threshold by utilizing three times the prior standard deviation, denoted as $\sigma$. In contrast, the non-impact function method establishes the discrimination threshold as three times the sample standard deviation, as outlined in Formula (2). The methodology presented in this study utilizes a constant $C_H=1.7$, with a sliding window length set at $n=50$. It is important to note that all three methods operate under identical conditions, differing solely in their respective discrimination thresholds.

In the field experiment, the algorithm is compiled and incorporated into the primary control software. We document the external guidance data that is received and processed in real-time, subsequently analyzing the algorithm’s actual performance. The primary control software is responsible for operating the theodolite^28^, facilitating communication with the multi-channel composite tracker on the machine via high-speed LAN or serial port, and establishing a connection with the remote central machine through Ethernet. Serving as the system’s direct control software, the primary control software enables monitoring, configuration, detection, and debugging of the entire equipment.

#### Simulation experiment

**Fig. 3:**
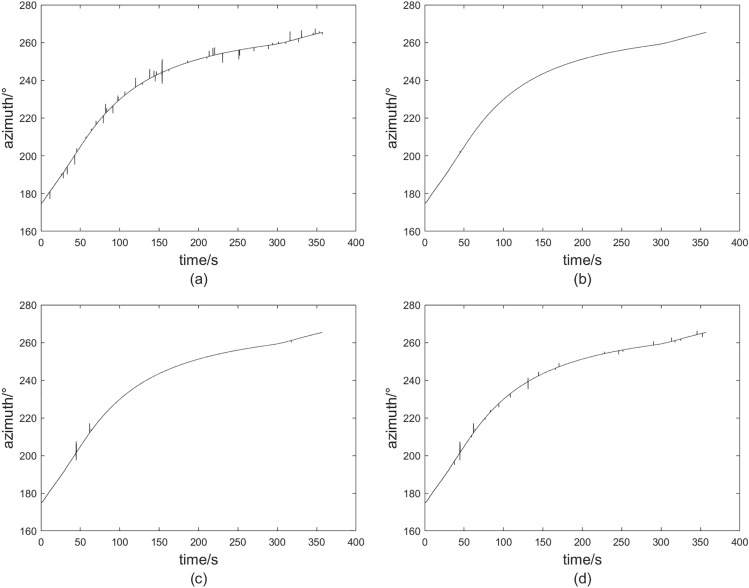
The outlier processing results of the three methods. (**a**) Data with outliers. (**b**) Result of fixed threshold method. (**c**) Result of non-impact function method. (**d**) Result of dynamic threshold.

Fifty isolated outlier points and five continuous outlier points, with random lengths ranging from 5 to 10, were incorporated into the dataset. The outcomes of the guidance data subsequent to the inclusion of these outliers, along with the results from the three methodologies, are presented in Fig. 3.

The systematic errors associated with the three outlier processing methods have been computed^29,30^, and the findings are presented in Table 2.

**Table 2:** Comparison of the system error.

Method	Fixed threshold	Non-impact function	This paper
System error	0.3070	0.2127	0.0045

**Fig. 4:**
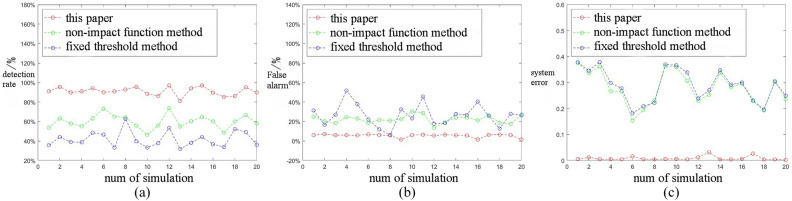
Processing results of simulations. (**a**) Comparison of detection rate. (**b**) False alarm rate comparison. (**c**) System error comparison.

In our experiment, we conducted the simulation twenty times while maintaining consistent conditions for outliers. We define the discrimination detection rate as the ratio of the number of outliers identified for elimination to the total number of outliers present. Additionally, we define the false alarm rate as the difference between the total number of eliminations and the number of outliers eliminated, divided by the total number of eliminations. The comparative analysis of the detection rate, false alarm rate, and system error for the three methodologies is illustrated in Fig. 4.

Table 3 shows the comparison between the rejection rate and false alarm rate of the three methods and the average MSE mean.

**Table 3:** Comparison of the average values of the indicators for multiple simulations.

Method	Fixed threshold	Non-impact function	This paper
Rejection average	0.3974	0.6013	0.8937
Average false alarm rate	0.3045	0.1047	0.0251
MSE mean	0.1116	0.0988	0.0052

#### Outfield experiment

**Fig. 5:**
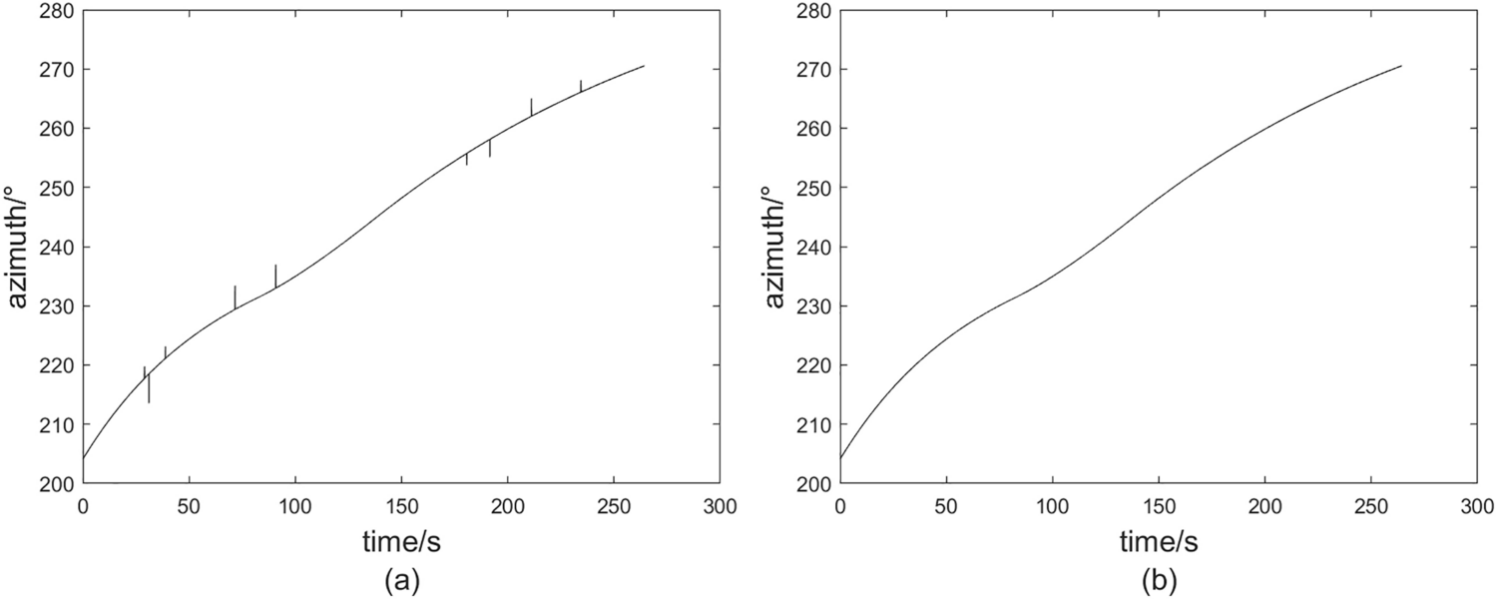
Outfield data processing results. (**a**) Guide data. (**b**) Outlier elimination.

In the context of a specific outfield experiment, we employ the outlier elimination technique to analyze the external guide data, as illustrated in Fig. 5.

The experimental findings indicate that the outlier elimination technique utilizing a dynamic threshold is effective in differentiating between isolated outliers and speckle outliers, achieving a detection rate exceeding 80% and exhibiting the lowest false alarm rate. In contrast, among the three evaluated methods, the outlier discrimination approach employing a fixed threshold demonstrates the lowest detection rate and lacks flexibility, as its efficacy is entirely reliant on a priori standard deviation. The outlier discrimination method based on the non-impact function employs Formula (2) to compute the sample standard deviation, which is contingent upon each individual sample point without differentiation. In instances where consecutive outliers are present within the sliding window, the estimated sample standard deviation can significantly diverge from the actual variance of the normal points, leading to the failure to detect numerous outliers. Regarding the false alarm rate comparison, the method proposed in this study effectively differentiates between normal and invalid values; thus, even if multiple points are classified as abnormal, the method can still utilize normal points to estimate the sample variance, allowing for timely dynamic adjustments of the threshold. Conversely, the estimation outcomes of the fixed threshold method and the non-impact function method are unable to adapt dynamically to the data characteristics, resulting in increased false alarms and misclassifications.

### Interpolation algorithm

The experiment is conducted in accordance with a specific outfield experimental task within a designated naval range. The primary control software acquires guidance data at a frequency of 20 Hz from the central station, executes coordinate transformations, and subsequently transmits real-time interpolation data at a frequency of 100 Hz to the servo system. For this study, we establish $L = 4$, a *threshold* value of 0.2, and a *constant* value of 0.0005.

**Fig. 6:**
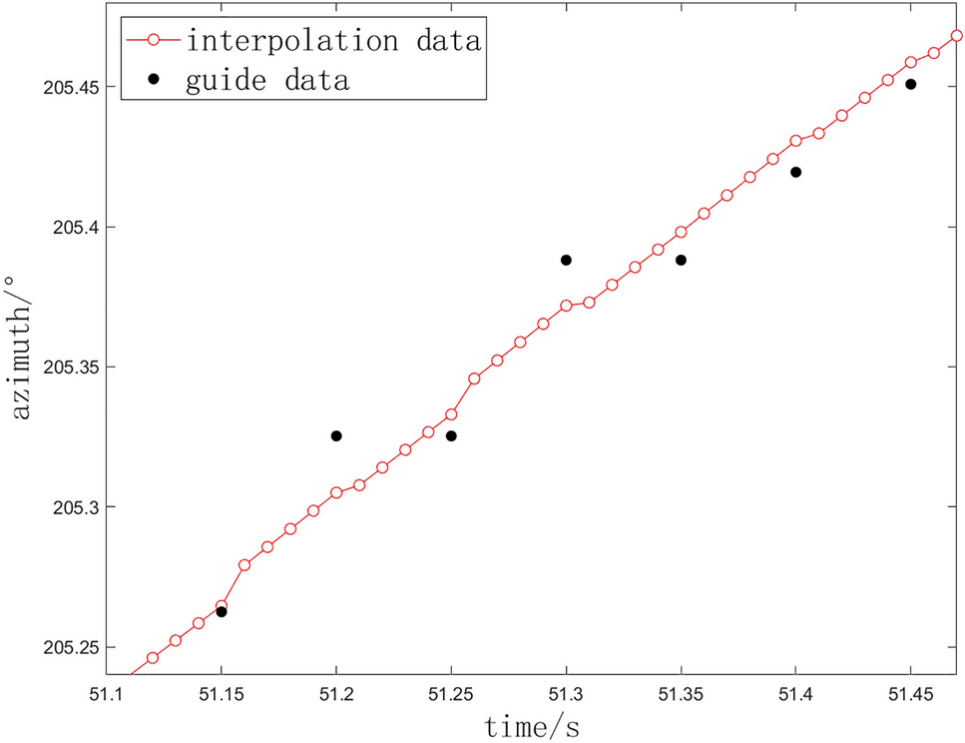
The interpolation results of azimuth angle for slight stuck points.

For the situation with slight stuck points, taking azimuth angle as an example, the external guidance received data and real-time interpolation data are shown in Fig. 6.

The red dots depicted in the figure represent the interpolation data generated by the methodology presented in this study, while the black ones signifie the original external guidance value. The interpolation data effectively captures the overarching trend of the data while preserving a smooth and coherent structure. In instances where there is minimal or no obstruction, the algorithm determines that the two interpolations can remain consistent; consequently, it directly employs the output from the linear least squares method as the result for the current interpolation.

**Fig. 7:**
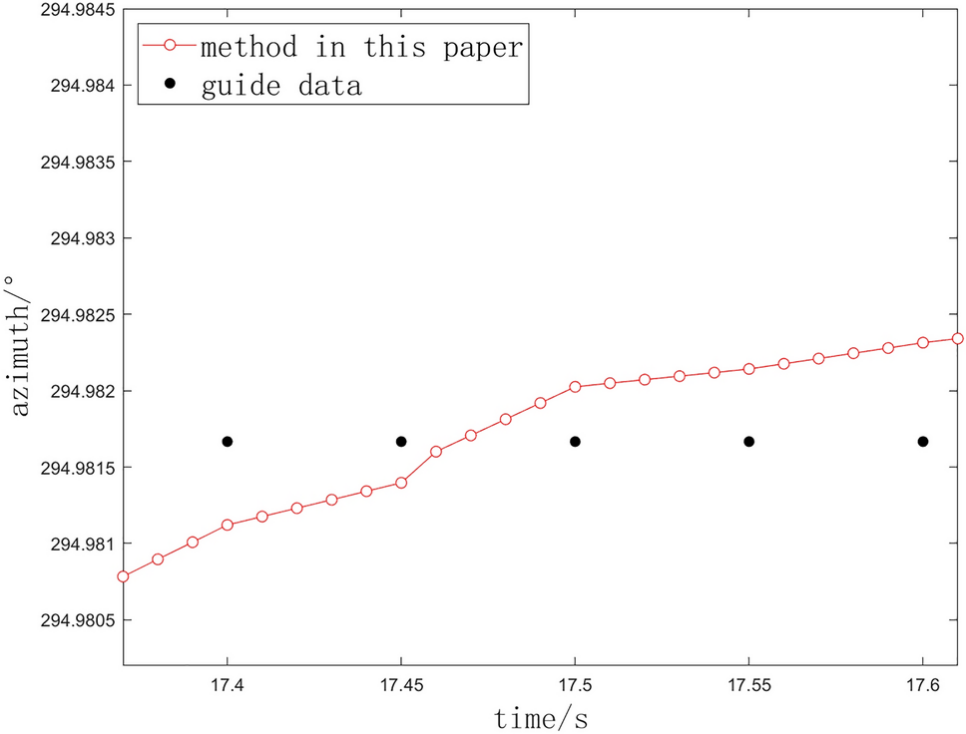
Interpolation for stuck points. (**a**) Method in this paper. (**b**) Comparison with linear least squares. (**c**) Comparison with Newton interpolation.

**Fig. 8:**
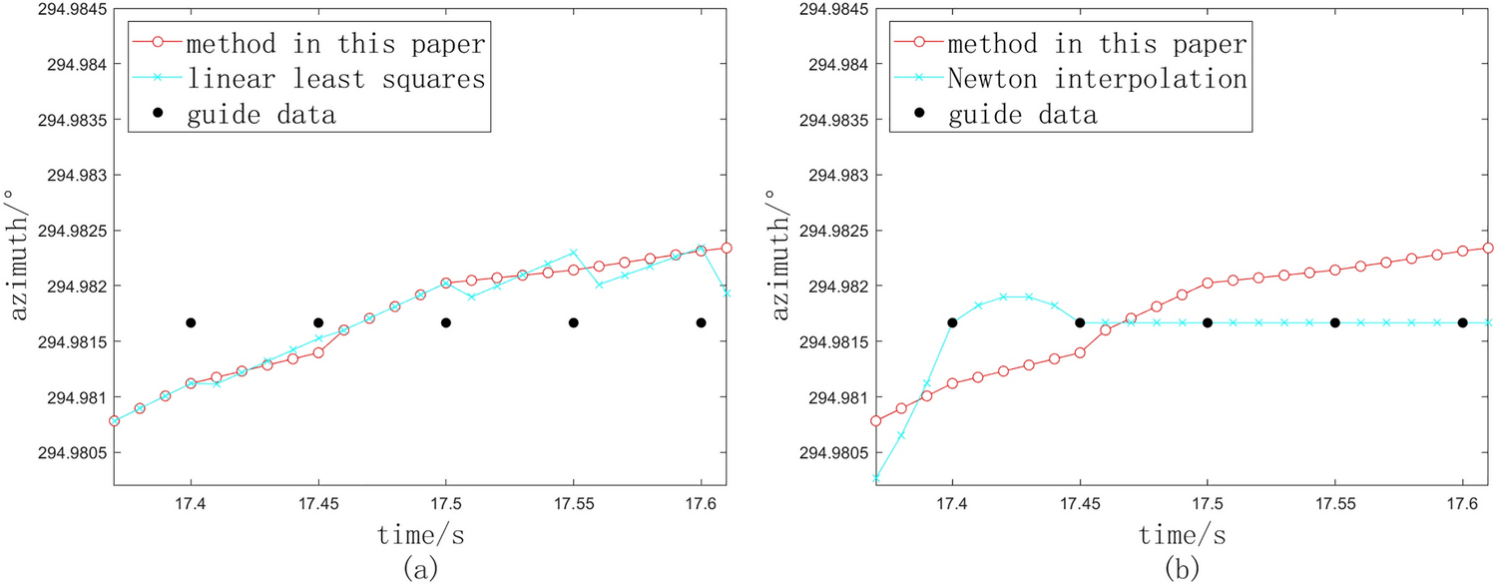
Interpolation for stuck points. (**a**) Method in this paper. (**b**) Comparison with linear least squares. (**c**) Comparison with Newton interpolation.

In the context of a specific task involving significant guidance data constraints, three distinct methodologies have been employed. As an illustration, the azimuth angle is examined, and the comparative analysis of the processing outcomes under conditions of severe data obstruction is presented in Figs. 7 and 8.

The blue dots depicted in Fig. 8a and b are the calculation results of linear least squares method and Newton interpolation method respectively, and the red dots are the interpolation results of our method. Because the algorithm adaptively judges whether the interpolation points are consistent, even if the theodolite moves slowly according to the interpolation results, each interpolation point can continue to increase or decrease according to the trend based on the previous interpolation point, which will not cause the photoelectric theodolite to rotate in reverse and start and stop frequently. The interpolation data generated by our method is smooth, and can be steady connected with the previous interpolation data.

We conduct a further examination of the angular velocity at the interpolation data points. It is observed that when the slope transition at each interpolation data point is more gradual, the variation in angular velocity approaches zero. We denote the absolute value of angular velocity as $K_i$ and the change in angular velocity as $\Delta K_i$.

12 $$\begin{aligned} K_i = \frac{Z_{i+1} - Z_i}{\Delta t} \end{aligned}$$

13 $$\begin{aligned} \Delta K_i = |K_i - K_{i-1}| \end{aligned}$$

Table 4 presents the cumulative values of azimuth interpolation data points, denoted as $\Delta K_i$, for the three methodologies employed in this experimental task.

**Table 4:** Comparison of the sum of interpolated data points.

Interpolation method	Linear least squares	Newton interpolation	This paper
$\Delta K_i$	79.040	41.440	22.829

The analysis indicates that the variation in angular velocity among the interpolation data points presented in this study is significantly reduced and exhibits a more gradual change. This characteristic is advantageous for the safeguarding of the servo system.

## Conclusion

This paper presents a real-time smoothing algorithm for external guidance data, utilizing an adaptive dynamic threshold. The algorithm encompasses both outlier processing and interpolation techniques.

1. The dynamic threshold-based outlier processing algorithm employs the influence function to derive the formula for calculating sample variance. This approach facilitates more precise outlier elimination and addresses issues related to inaccurate threshold settings and elevated false alarm rates. Experiments show that compared with the fixed threshold and the five-point extrapolation method without influence function, the outage rejection percentage of outage processing algorithm is increased by 127.92% and 48.97% respectively, the average is above 85%, and the false alarm rate is reduced by 91.76% and 76.05% respectively.The method proposed in this paper can better dynamically adapt to the data characteristics by evaluating the external guiding value points participating in the sample variance calculation.
2. The adaptive interpolation-based processing algorithm is capable of assessing data consistency in an adaptive manner. It employs various strategies based on the severity of data stagnation, allowing it to adjust to varying levels of obstruction. The interpolation method introduced is designed to produce data that is free from jitter and discontinuities, and experimental results demonstrate an excellent smoothing effect.Compared with linear least squares and Newton interpolation, the sum of angular velocity gradients of the interpolation algorithm is reduced by 71.12% and 44.91% respectively.The angular velocity variation of the interpolated data points in this paper is obviously smaller, and the change is the most gentle, which is more conducive to protecting the servo system.

The methodology presented in this paper has been effectively implemented on a naval range measurement platform, successfully meeting the requirements of real-time, accuracy and smoothness of external guidance data processing. This method based on dynamic threshold and smooth interpolation can be applied to other fields requiring real-time data processing and outlier detection, such as radar data processing, satellite trajectory measurement, etc., and has a wide range of practical applications.

## Future work

The outfield processing and interpolation algorithms in this paper aim at external guidance information, and other data sources, such as theoretical trajectory, can be added in the future, so that the algorithm in this paper can be applied to various working modes of the theodolite. For example, when the external guidance mode is switched to other modes, the connection of the theodolite can be ensured, so that the theodolite can have a higher stable tracking ability. On the other hand, this method based on dynamic threshold and smooth interpolation can be applied to other fields requiring real-time data processing and anomaly detection, such as radar data processing and satellite trajectory measurement, and has a wide range of practical applications.

## Data Availability

All data generated or analysed during this study are included in this published article.
